# Protective Effect of Quercetin on the Development of Preimplantation Mouse Embryos against Hydrogen Peroxide-Induced Oxidative Injury

**DOI:** 10.1371/journal.pone.0089520

**Published:** 2014-02-21

**Authors:** Sha Yu, Hui Long, Qi-feng Lyu, Qin-hua Zhang, Zhi-guang Yan, Hong-xing Liang, Wei-ran Chai, Zheng Yan, Yan-ping Kuang, Cong Qi

**Affiliations:** 1 Department of Gynecology, Shuguang Hospital Affiliated to Shanghai University of Traditional Chinese Medicine, Shanghai, People's Republic of China; 2 Department of Assisted Reproduction, Shanghai Ninth People's Hospital Affiliated Shanghai Jiao Tong University School of Medicine, Shanghai, People's Republic of China; Justus-Liebig-Universität, Germany

## Abstract

Quercetin, a plant-derived flavonoid in Chinese herbs, fruits and wine, displays antioxidant properties in many pathological processes associated with oxidative stress. However, the effect of quercetin on the development of preimplantation embryos under oxidative stress is unclear. The present study sought to determine the protective effect and underlying mechanism of action of quercetin against hydrogen peroxide (H_2_O_2_)-induced oxidative injury in mouse zygotes. H_2_O_2_ treatment impaired the development of mouse zygotes *in vitro*, decreasing the rates of blastocyst formation and hatched, and increasing the fragmentation, apoptosis and retardation in blastocysts. Quercetin strongly protected zygotes from H_2_O_2_-induced oxidative injury by decreasing the reactive oxygen species level, maintaining mitochondrial function and modulating total antioxidant capability, the activity of the enzymatic antioxidants, including glutathione peroxidase and catalase activity to keep the cellular redox environment. Additionally, quercetin had no effect on the level of glutathione, the main non-enzymatic antioxidant in embryos.

## Introduction

A number of studies have demonstrated that blastocyst transfers can offer higher rates of pregnancy and live birth than the transfer of cleavage-staged embryos [1], [2]. Although many advances have been made in cell culture technique, the proportion of embryos that develop to the blastocyst stage is still variable [3], [4]. Oxidative stress, a cellular condition caused by the accumulation of reactive oxygen species (ROS), is thought to contribute significantly to defective embryo development [5]–[8]. Additionally, oxidative stress was correlated with the pathological processes of female infertility induced by maternal aging, endometriosis and other unexplained factors [9].

ROS, such as superoxide anion radicals (O_2_·^−^), hydrogen peroxide (H_2_O_2_) and hydroxyl radicals (·OH), are inevitably generated during aerobic metabolism and play important roles in many physiological processes, including gamete maturation, fertilization and embryo growth [10]. However, some exogenous oxidants, such as H_2_O_2_, will result in the overproduction of ROS, which may induce multiple cellular damages, including lipid peroxidation, nuclear DNA strand breaks, and mitochondrial alteration, consequently disturbing the development of preimplantation embryos *in vitro* [11]–[13]. For example, in obese mice, compromised oocyte and zygote mitochondrial metabolism induced by oxidative stress were associated with significant developmental impairment [14]. Liu *et al.* demonstrated that accumulation of ROS induced developmental arrest of embryos by altering mitochondrial activity and structures [11]. However, low concentrations of ROS were shown to reduce DNA fragmentation and improve the developmental potential of porcine embryos [13]. These reports suggest that high ROS level is harmful for preimplantation embryo development.

Several defense mechanisms are present in both embryos and their environment to remove excess ROS. As non-enzymatic antioxidants, vitamins (e.g. A, C, and E) and glutathione (GSH) are able to react with ROS and maintain appropriate levels. In addition, enzymatic defense systems, including superoxide dismutase (SOD), glutathione peroxidase (GPx) and catalase (CAT), also suppress oxidative injury efficiently and weaken the damage caused by ROS [15]. However, if the level of ROS goes beyond to the capacity of the anti-oxidative defense systems, oxidative stress will occur. Therefore, many antioxidants are widely used to reduce excess ROS to promote embryo development [16]–[18]. For example, the addition of glutamine and hypotaurine to porcine embryos promoted development by reducing the content of H_2_O_2_ [17]. Moreover, the presence of Vitamin E in culture may improve buffalo embryo developmental competence under oxidative stress [18].

Quercetin (3, 3′, 4′, 5, 7-pentahydroxyflavone) is one of the most ubiquitous flavonoids and is found in many Chinese herbs, fruits and red wine [19], [20]. Accumulating evidence has shown that quercetin possesses efficient antioxidant activity [21]–[24]. Animal experiments suggest that quercetin protected the brain, liver, and other tissues against oxidative stress induced by ischemia-reperfusion injury, toxic compounds and other factors [22]–[24].

The antioxidative capacity of quercetin is attributed to its chemical structure [20]. The spatial arrangement and total number of hydroxyl groups located at the B-ring have been postulated to account for the antioxidant potency of quercetin [20]. In addition to the direct antioxidative action, quercetin also protects mitochondrial function [25] and regulates enzyme antioxidative defense systems [26]–[28]. It has been reported that quercetin protected Caco-2 cells against indomethacin-induced mitochondrial dysfunction [25]. Moreover, quercetin also regulates the activities of GPx and CAT in chicken embryos [27] and SOD and CAT activities in diabetic rats against oxidative injury [28]. Although an antioxidant effect of quercetin has been reported in some mammalian cell models [29], [30], there is currently no research regarding the effect of quercetin on preimplantation embryo development.

The aim of the present study was to determine whether quercetin have the ability to protect preimplantation mouse embryos against oxidative injury and to explore the underlying cellular and biochemical mechanisms. For the first time, we show an embryo-protective effect of quercetin against oxidative injury. Furthermore, improvement of embryo viability was associated with the decreased ROS level, stable mitochondrial function and modulation of the activity of enzymatic antioxidants.

## Materials and Methods

### Ethical statement

Experiments with animals were performed in accordance with the National Institutes of Health Guide for the Care and Use of Laboratory Animals, and approved by the ethical committee of Shanghai Jiao tong University (Permit Number: SYXK 2007–0007).

### Animals

Kunming mice (4–6 weeks old) used for these experiments were purchased from SLAC Laboratory Animal Co. Ltd (Shanghai, PRC). All animals were housed under controlled conditions, with temperatures between 22 and 24°C and a 12 h light/12 h dark photoperiod.

### Reagents and media

All reagents were purchased from Sigma (St. Louis, MO, USA) unless otherwise stated. Potassium simplex optimized medium (KSOM; Merck Millipore MR-106-D; Billerica, MA, USA) was used as the standard medium for culturing embryos. All manipulations of embryos were performed using mHTF-SSS medium [90% (*v*/*v*) modified human tubal fluid medium (mHTF; Irvine Scientific; NP Waalwijk, NED) and 10% (*v/v*) serum substitute supplement (SSS; Irvine Scientific; Santa Ana, CA, USA)]. Dimethyl sulfoxide (DMSO) was used as vehicle for quercetin. H_2_O_2_ (China National Medicine Co. LTD; Beijing, PRC) was diluted in KSOM for induction of oxidative injury.

### Collection of mouse zygotes

Kunming female mice were super-ovulated by intraperitoneal (i.p.) injection of 10 IU pregnant mare serum gonadotropin (PMSG; Sansheng; Ningbo, Zhejiang, PRC) and followed 46–48 hours later by injection of 10 IU human chorionic gonadotropin (hCG; Sansheng; Ningbo, Zhejiang, PRC). Super-ovulated females were then mated individually with fertile Kunming males. The following morning, females with vaginal plugs were selected and humanely sacrificed by cervical dislocation at 21–22 h after hCG injection. Zygotes (Day 0.5) were collected from the oviduct ampullae and freed from cumulus cells by a 3–5 min treatment with 70 μg/ml bovine testicular hyaluronidase in mHTF-SSS medium. After thoroughly rinsing the recovered zygotes in mHTF-SSS, cumulus-free zygotes were maintained in 20 μl droplets of KSOM, covered with mineral oil at 37°C in a humidified atmosphere of 5.2% CO_2_.

### Quercetin and hydrogen peroxide treatment

Two-pronuclear embryos were selected for experiments. To determine the oxidative damage, zygotes were exposed to different concentrations of H_2_O_2_ (0–75 μM) for 15 minutes, washed extensively, and then cultured in KSOM until day 5.5. For dose–response study of quercetin, zygotes were cultured in KSOM medium with different concentrations of quercetin (0–50 μM) until day 5.5. To determine the protective effect of quercetin against oxidative stress, zygotes were pre-incubated with quercetin (0, 1, 5 or 10 μM) in DMSO or DMSO alone (0.1% or 0.01%) for 3 h. After pre-incubation, quercetin or DMSO was maintained and H_2_O_2_ was added for 15 min. Then, washed out and embryos were cultured in media with quercetin or DMSO again until day 5.5. Zygotes of the control group were culture in KSOM continuously. Cleavage and embryo development were examined every 20–24 h until 5.5 days.

### TUNEL assay

Nuclear DNA fragmentation in blastocysts was assessed using a terminal deoxynucleotidyl transferase (TdT)-mediated dUTP nick end-labeling assay (TUNEL), according to the manufacturer's instructions (APO-BrdU TUNEL Assay Kit; Invitrogen, USA). After TUNEL assay performed, embryos were washed and counterstained with Hoechst 33342 (5 μg/ml; Molecular Probes) for 5 minutes in the dark at RT. Fluorescence was detected using an inverted uorescence microscope and photographed by a digital camera (Nikon; Tokyo, Japan). Quantification of TUNEL positive cells and total cell numbers in blastocysts was performed using Image-Pro Plus 5.1 software (Media Cybernetics).

### Determination of ROS products

To measure ROS levels, after exposing cumulus-denuded zygotes to H_2_O_2_, each group was incubated in KSOM supplemented with dichlorodihydrofluorescein diacetate (DCFH-DA; 2 μM, Molecular Probes) for 30 min at 37°C as previously described [31].

### Detection of Mitochondrial Membrane Potential (MMP)

Mitochondrial membrane potential (△Ψm) was determined by staining zygotes with 5, 5′, 6, 6′-tetrachloro-1, 1′, 3, 3′-tetraethylbenzimidazolyl-carbocyanine iodide (JC-1; Beyotime Biotechnology; Haimen, Zhejiang, PRC). After exposing to H_2_O_2_, cumulus-denuded zygotes were incubated in KSOM supplemented with JC-1 (1.25 μM) for 20 min at 37°C as previously described [11]. Then washed and immediately examined using a fluorescent microscope (Nikon, Tokyo, Japan). In the presence of a high MMP, JC-1 forms J-aggregates that emit red fluorescence, while JC-1 monomeric form emits green fluorescence at low MMP. The average red and green pixel intensity was determined and MMP expressed as the ratio of red intensity to green intensity.

### Total antioxidant capability assay

The total antioxidant capability (T-AOC) of embryos was measured with a commercial kit (Nanjing Jiancheng Biochemistry Reagent Co; Nanjing, Jiangsu, PRC) based on the ferric reducing/antioxidant power (FRAP) method [32]. After exposure to H_2_O_2_, a total of 30–50 cumulus-free zygotes from each group were lysed and vortexed for 5 min, then frozen at −80°C and thawed at 37°C three times. The mixture was centrifuged at 10,000 rpm for 10 min at 4°C and put on ice. Then, according to the manufacturer's instructions, the samples were added into the tubes containing the substrate o-phenanthroline and incubated at 37°C for 30 min. The embryo lysate reduces Fe^3+^ to Fe^2+^, and Fe^2+^ binds with o-phenanthroline, which produces a visible chelating agent. The absorption of the mixture was measured at 520 nm using a spectrophotometer. T-AOC was expressed in Units/ml and one Unit was defined as an increase in absorbance (A520) of 0.01/min/ml lysate at 37°C.

### GPx activity

The activity of GPx was measured using the GSH-Px Assay Kit (Nanjing Jiancheng Biochemistry Reagent Co; Nanjing, Jiangsu, PRC) according to the manufacturer's instructions through a coupled assay using H_2_O_2_ and dithio-bis-nitrobenzoic acid (DTNB) [32]. Absorbance was recorded at 412 nm. The GPx activity was expressed as Units/ml and one Unit was defined as a decrease in GSH concentration of 1mmol/L/min after subtraction of non-enzymatic mode.

### CAT activity

The embryo extract was collected as above and CAT activity was assayed with a commercial assay kit (Beyotime Biotechnology; Haimen, Zhejiang, PRC) using H_2_O_2_ as a substrate [33]. The absorbance was measured maximally at 520 nm and expressed in Units/mg protein. The protein concentration was measured with a Pierce BCA protein assay kit (Thermo, USA).

### GSH level

The concentrations of total glutathione were determined by a spectrophotometer using a commercial assay kit (Beyotime Biotechnology; Haimen, Zhejiang, PRC) as previously described [31]. According to the manufacturer's instructions, the collected embryo supernatant was mixed with the working buffer (4.3 μl glutathione reductase, 2.2 μl DTNB and 150 μl buffer solution). After reacting for 5 min at RT, 50 μl of 0.16 mg/ml NADPH was added and the absorbance was monitored at 412 nm. Standards (15, 10, 5, 2, 1 and 0.5 μM) of GSH and a blank sample lacking GSH were also assayed. The activity of GSH was expressed as pmol per embryo.

### Statistical analysis

Results were obtained from at least three independent experiments and analyzed using SPSS software (SPSS Inc., Chicago, IL, USA). The differences between means were calculated with one-way analysis of variance (ANOVA) followed by Fisher's least significant difference (LSD). Values are expressed as the mean ± standard error of the mean (SEM). Data expressed as percentages were analyzed using *x* ^2^-test. *P*<0.05 were considered statistically significant.

## Results

### Quercetin protected the development of mouse preimplantation embryos against oxidative stress induced by H_2_O_2_

First, the dose-effect of quercetin on embryonic development was investigated. Table S2 showed that quercetin at 1 μM, 5 μM, 10 μM, had no significant effect on the embryonic development, while quercetin at 50 μM produced a reduction in the rates of morula, blastocyst formation, and hatched (*P*<0.001). To induce oxidative stress, H_2_O_2_ was used to treat mouse zygotes. When mouse zygotes were exposed to H_2_O_2_ at 35 μM, the rates of blastocyst formation and hatched decreased significantly (*P*<0.01) and exhibited a dose-dependent decline (Table S1). Then, the effect of quercetin on the development of preimplantation embryos was evaluated following the treatment of zygotes with 35 μM H_2_O_2_. As shown in Table 1, low-dose quercetin exposure (1 μM) seemed to provide mild protection as indicated by an increase in the percentage of embryos that reached the morula stage when compared with H_2_O_2_ treatment alone. Furthermore, with a concentration of quercetin up to 5 μM, blastocyst formation and hatched rate were recovered to levels of the control group. When zygotes were exposed to high-dose quercetin (10 μM), the rate of embryos reaching the blastocyst stage were extensively recovered as well. However, quercetin at 10 μM exposure only increased the hatched rate slightly as compared with the H_2_O_2_ group (35.165% vs. 26.374%) and there was no significant difference between them (Table 1).

**Table 1:** Protective effect of quercetin on the development of preimplantation mouse embryos against H_2_O_2_-induced oxidative injury.

				Zygotes developing to: n (% of A)			
Groups	Conc. of H_2_O_2_ (μM)	Conc. of Que (μM)	No. of zygotes (A)	Two–cell stage	Morula stage	Blastocyst stage	Hatched stage
Control	0	0	274	272 (99.270)	241 (87.956)	218 (79.562)	126 (45.985)
H_2_O_2_	35	0	182	164 (90.110)^ ***^	117 (64.286)^***^	99 (54.396)^***^	48 (26.374)^***^
H_2_O_2_+Que	35	1	196	186 (94.898)^ **^	150 (76.531)^**,^ #	118 (60.204)^***^	62(31.633)^**^
	35	5	191	189 (98.953)	163 (85.340)^###^	146 (76.440)^###^	87(45.550)^###^
	35	10	182	179 (98.352)	152 (83.516)^###^	144 (79.121)^###^	64(35.165)*

Differences between the groups were calculated using the *x* ^2^-test. Que: quercetin.

^*^ *P*<0.05, ^**^ *P*<0.01, ^***^ *P*<0.001 vs. the control group.

# *P*<0.05, ^##^ *P*<0.01,^ ###^ *P*<0.001 vs. the H_2_O_2_-treated group.

Additionally, to confirm the antioxidative effect of quercetin excluding the functional interference of DMSO, the vehicle employed to dissolve quercetin, the development of mouse embryos exposed to DMSO was investigated. As shown in Table S3, 0.01% DMSO treatment, the concentration used to dissolve quercetin in our experiment, had no significant effect on the rates of blastocyst formation and hatched, as compared with the H_2_O_2_ treated group (P = 0.23, and P = 0.16). Moreover, 0.1% DMSO treatment augmented the impairment of embryo development under oxidative stress.

**Figure 1:**
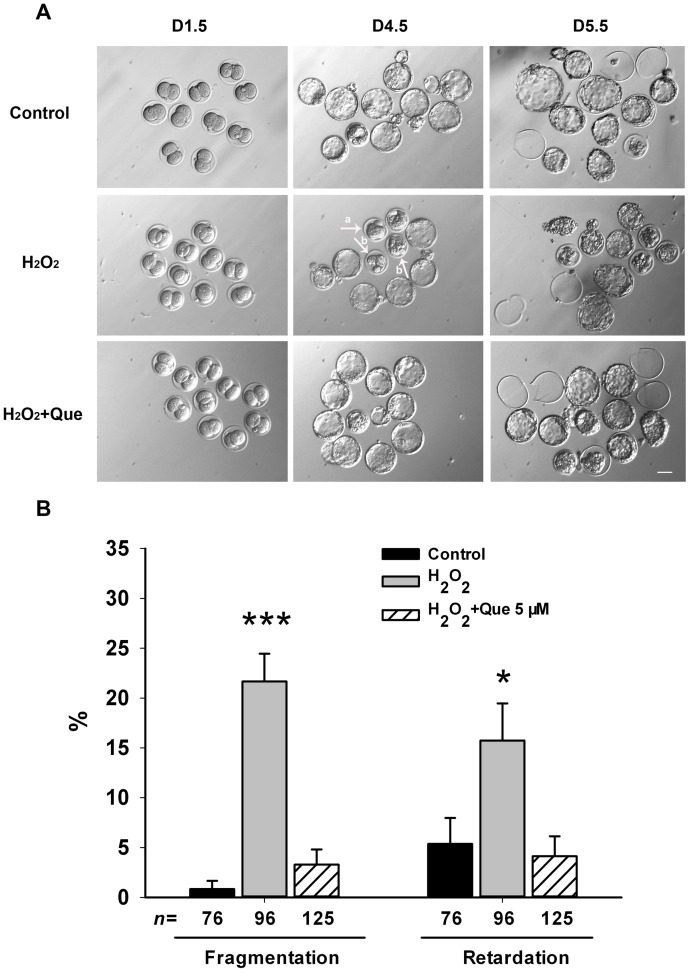
Quercetin prevented H_2_O_2_-induced fragmentation and retardation in blastocysts. (A) Representative images of pre-implantation embryos cultured in the presence or absence of quercetin (Que) under H_2_O_2_-induced oxidative stress. Zygotes were pre-treated for 3 h with 5 μM Que followed by addition of 35 μM H_2_O_2_ for 15 min, and subsequently cultured in presence of Que until day 5.5. The morphology of pre-implantation embryos was imaged at D1.5 (2-cell), D4.5 (blastocyst) and D5.5 (hatched) after fertilization. In the H_2_O_2_-treated group, developmental retardation (arrow a) and fragmentation (arrow b) were observed in blastocysts. Normal development and high embryo quality were apparent in the Que-treated group at 5 μM. Bar, 50 μm. (B) Quantification of fragmented and retarded blastocysts. Data are presented as the mean ± SEM from three independent experiments. N shows the number of embryos and ^*^ *P*<0.05; ^***^ *P*<0.001, compared with the control group (One-way ANOVA).

Furthermore, the analysis of morphology revealed that treatment of zygotes with H_2_O_2_ induced fragmentation and growth retardation at day 4.5 *in vitro*, while embryos from the quercetin and control groups exhibited much less (Fig. 1A). Quantification analysis showed that there was a significant increase in the rates of fragmented and retarded embryos in the H_2_O_2_-treated group and that quercetin prevented this effect (Fig. 1B). These data suggested that quercetin has a positive effect on pre-implantation embryo development in detrimental culture conditions of oxidative stress.

### Quercetin decreased apoptosis in blastocysts developed from zygotes exposed to oxidative stress

**Figure 2:**
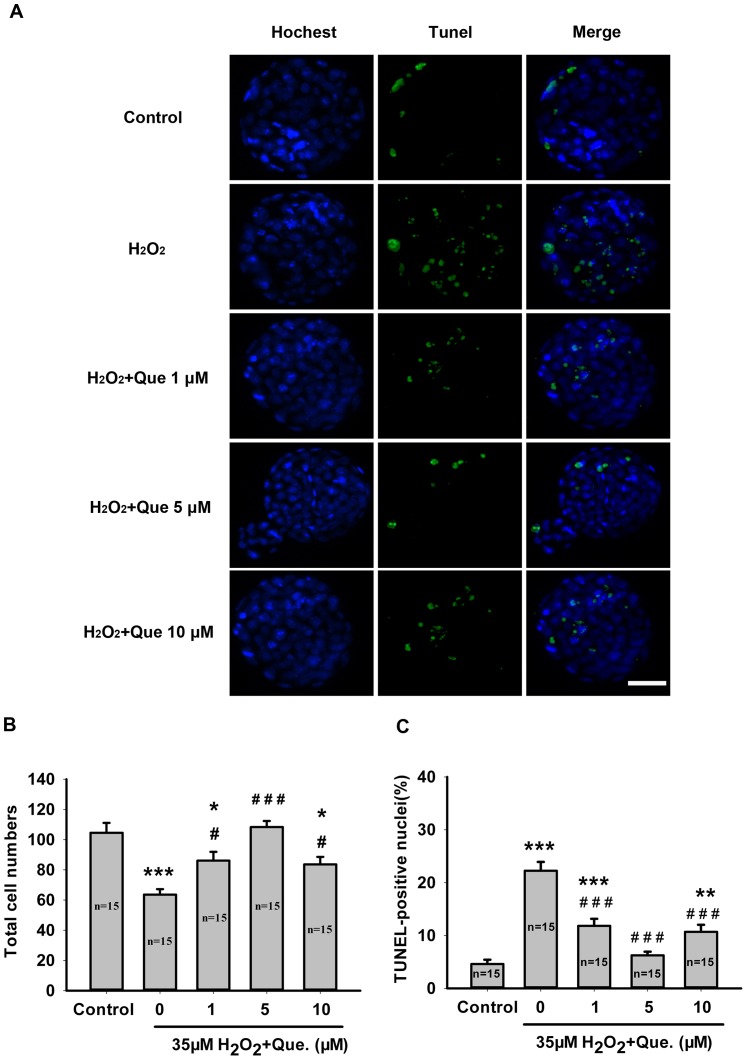
Quercetin attenuated H_2_O_2_-induced apoptosis in blastocysts. (A) Representative images of TUNEL positive cells in mouse blastocysts after treatment with H_2_O_2_ or H_2_O_2_ plus Que during the zygote stage. TUNEL positive cells were stained with Alexa Fluor 488 dye and all nuclei were labeled with Hoechst 33342 (blue). Bar, 50 μm. (B) Quantification of the total cell numbers per blastocyst. (C) Percentage of TUNEL positive cells per blastocyst. Data are presented as mean ± SEM from three independent experiments. N shows the number of blastocysts. ^*^ *P*<0.05, ^**^ *P*<0.01, ^***^ *P*<0.001 vs. the control group; ^#^ *P*<0.05, ^##^ *P*<0.01, ^###^ *P*<0.001 vs. the H_2_O_2_-treated group (One-way ANOVA).

A TUNEL assay was used to determine the apoptosis rate in blastocysts developed from the H_2_O_2_-treated zygotes in the presence or absence of quercetin. As shown in Figure 2, H_2_O_2_ treatment induced apoptosis and decreased the total cell number in blastocysts in comparison with the control group (*P*<0.001). Under oxidative stress, quercetin minimized the negative influence of H_2_O_2_ on embryo development. Blastocysts developed from zygotes in the presence of all concentrations of quercetin (1, 5 and 10 μM) displayed normal morphology and had more cells than those treated only with H_2_O_2_ (Fig. 2A). Furthermore, the number of TUNEL positive cells decreased significantly in blastocysts derived from all concentrations of quercetin-treated groups as well (*P*<0.001, Fig. 2B,C), suggesting a protective effect of quercetin against apoptosis in blastocysts.

### Quercetin reduced H_2_O_2_ -induced intracellular ROS level in zygotes

**Figure 3:**
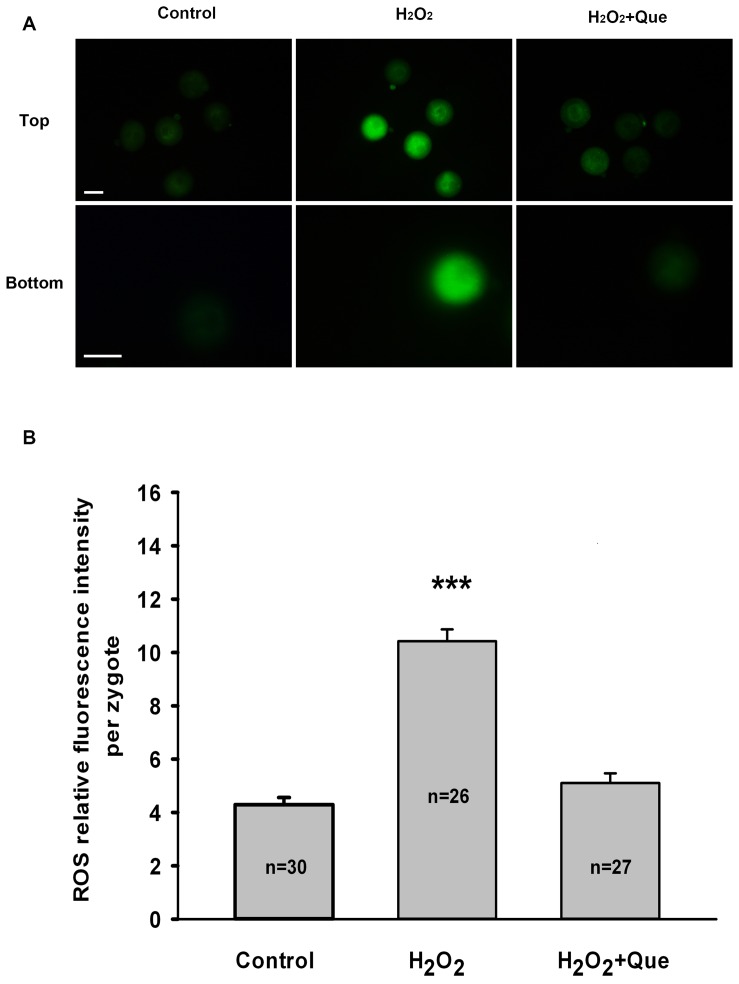
Quercetin decreases H_2_O_2_-induced intracellular ROS level in mouse zygotes. (A) Representative images of ROS levels in zygotes treated with H_2_O_2_ or H_2_O_2_ plus Que. The lower panels of images represent a magnified view of one zygote from the upper panels. Intracellular ROS were measured by the DCFH fluorescence (Green). Bar, 50 μm. (B) Quantification of relative fluorescence intensity of ROS per zygote. Data are presented as the mean ± SEM from three independent experiments. N shows the number of zygotes. ^***^ *P*<0.001, compared with the control group (One-way ANOVA).

To determine whether quercetin improves resistance to oxidative stress through regulation of ROS levels in embryos, ROS production in zygotes was measured using DCFH fluorescent reaction. When compared with the control group, the DCFH fluorescence intensity was much higher in the H_2_O_2_-treated group, whereas it weakened markedly in the presence of quercetin (Fig. 3A). The quantification analysis showed that quercetin exposure significantly decreased the level of intracellular ROS in zygotes (*P*<0.001, Fig. 3B), suggesting that quercetin can prevent an increase in ROS induced by H_2_O_2_.

### Quercetin prevented mitochondrial dysfunction induced by H_2_O_2_ in zygotes

**Figure 4:**
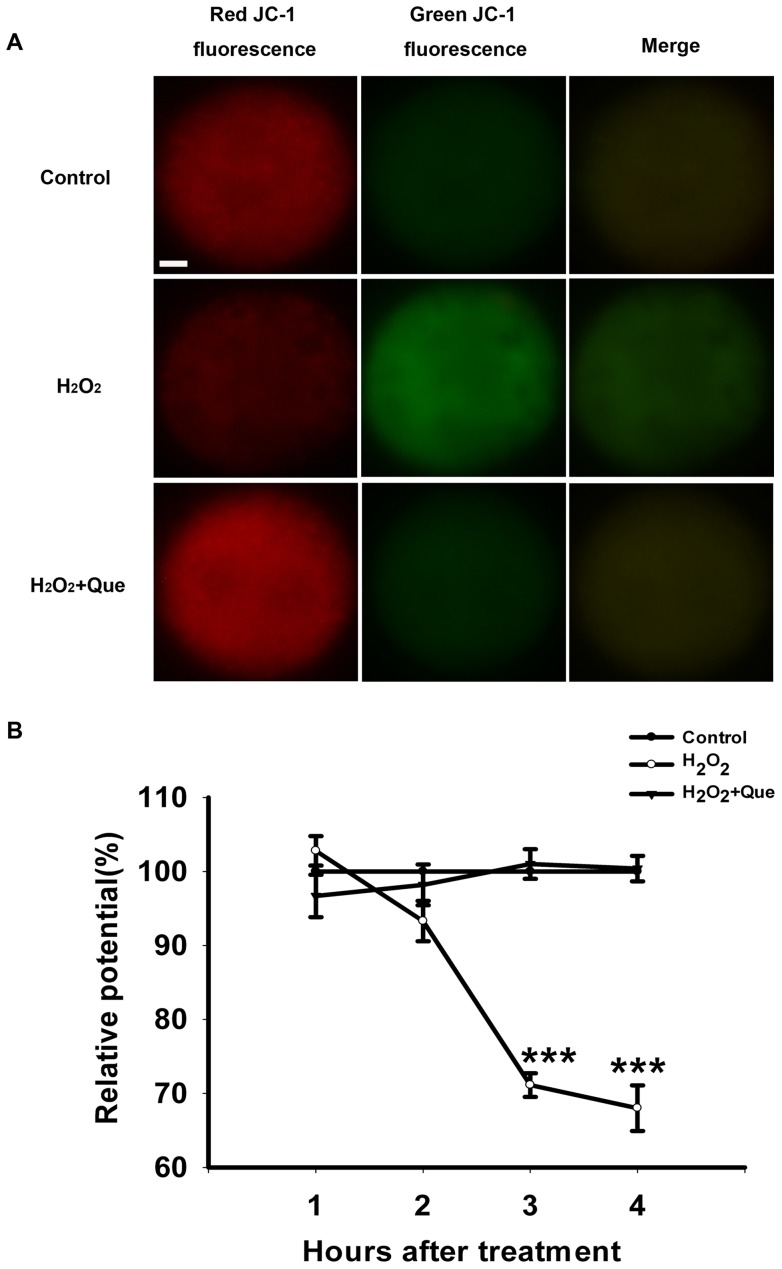
Quercetin prevented mitochondrial dysfunction induced by H_2_O_2_ in mouse zygotes. (A) Representative photomicrographs of inner mitochondrial membrane potential (MMP) in mouse zygotes 3 hours later after treated with H_2_O_2_ or H_2_O_2_ plus Que. Zygotes was stained with JC-1. Red fluorescence represented J-aggregates (high polarized mitochondria) and green fluorescence represented monomer form of JC-1 (low polarized mitochondria). Bar, 10 μm. (B) The ratios of intensity of red /green fluorescence (MMP) were analyzed from the images. The average of MMP from zygotes in the control group at each time point was set at 100%, and the MMP in treated zygotes were expressed relative to that of zygotes in the control group at each time point. MMP in zygotes declined 2 hours later after treatment of 35 μM H_2_O_2_ for 15 min and continued to drop over the subsequent hours. Quercetin can maintain MMP at the similar level of the control group. Data are presented as mean ± SEM from three independent experiments. ^***^ *P*<0.001 compared with the control group (One-way ANOVA, n = 15 for each group).

Since mitochondria are major organelles that produce ROS, and their malfunction will compromise embryo development [14], [34], we further explored MMP in zygotes at 1, 2, 3 and 4 h after H_2_O_2_-treatment. After exposure of zygotes to H_2_O_2_, the MMP depolarized rapidly, as shown by the enhanced green fluorescence and attenuated red fluorescence compared with the control group (Fig. 4A). The quantification of the ratio of fluorescence intensity (red/green), decreased nearly 30% after H_2_O_2_ exposure 3 hours later (*P*<0.001, Fig. 4B). However, quercetin treatment recovered the MMP to the similar level of the control group, suggesting that quercetin may maintain mitochondrial function against oxidative stress in zygotes.

### Quercetin protected preimplantation embryos from oxidative injury by sparing antioxidative enzymes

**Figure 5:**
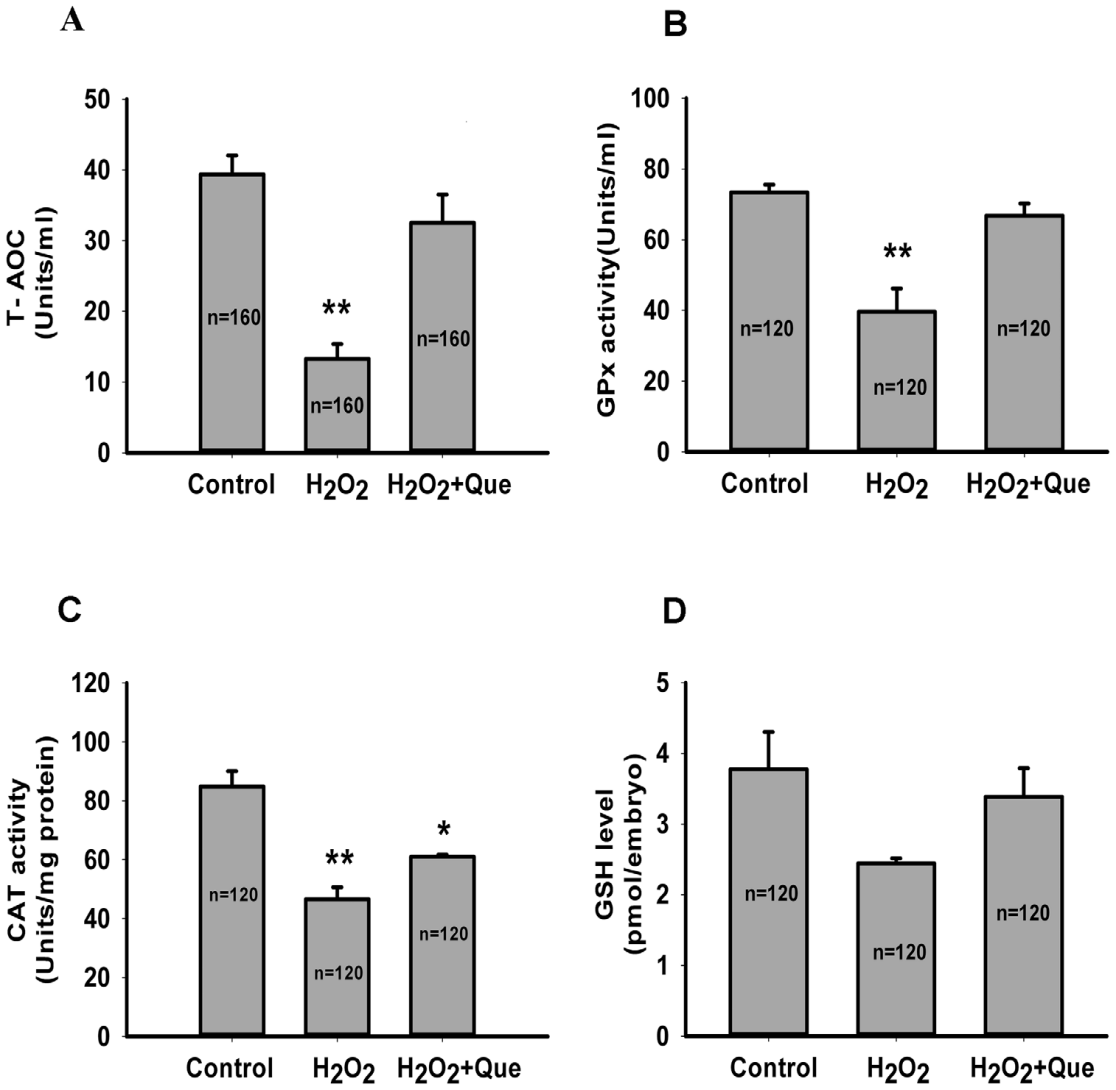
Quercetin attenuates H_2_O_2_-induced oxidative injury in mouse zygotes by sparing antioxidative enzymes. Zygotes were pre-treated for 3 h with 5 μM Que followed by addition of 35 μM H_2_O_2_ for 15 min, and then cultured in the presence of Que until day 5.5. (A) T-AOC, (B) GPx activity, (C) CAT activity and (D) GSH level were then measured. Data are presented as the mean ± SEM from at least three independent experiments and the number “n” in middle of each bar of the graphs indicates the total number of treated zygotes. ^**^ *P*<0.01, ^*^ *P*<0.05 compared with the control group (One-way ANOVA).

As H_2_O_2_ is a free radical generator and may lead to the decrease of antioxidant capacity in zygotes, we investigated the T-AOC in zygotes treated with H_2_O_2_ in the presence or absence of quercetin. Figure 5 shows that H_2_O_2_ treatment decreased T-AOC by approximately 66.3% (from 39.38±2.67 to 13.27±2.11 Units/ml) and quercetin was able to completely prevent this effect (32.52±4.00 Units/ml, Fig. 5A). In order to determine the effect of quercetin on antioxidant cellular defenses, we evaluated the enzymatic and non-enzymatic antioxidants in zygotes, including CAT, GPx and GSH. As compared with the control group, H_2_O_2_ treatment resulted in a significant decrease in the activities of GPx and CAT (*P*<0.01), whereas, quercetin exposure completely rescued GPx activity (*P*>0.05, Fig. 5B) and partially rescued CAT activity (Fig. 5C). In contrast, there was no significant difference among three groups in the level of GSH, the main non-enzymatic antioxidant in embryos (Fig. 5D, *P*>0.05). These data suggest that quercetin may protect preimplantation embryos from oxidative injury by sparing antioxidative enzymes.

## Discussion

During the development of preimplantation embryos *in vitro*, oxidative stress is one of the deleterious factors on fertilization and embryo quality [10]. The current study showed that quercetin was able to promote efficient development of mouse zygotes to the blastocyst stage in the presence of H_2_O_2_ and decrease apoptosis in blastocysts, suggesting quercetin had a protective effect against oxidative stress on preimplantation embryos.

The antioxidative capacity of quercetin has been shown in several other cell types, including neuronal cells [35] and spermatogonial cells [36], but this study is the first report to evaluate the effects of quercetin on the development of preimplantation embryos under oxidative stress. Our data showed that quercetin exposure greatly improved the quality of H_2_O_2_-treated embryos, indicated by increased blastocyst formation, reduced apoptosis and fewer incidences of fragmentation and developmental retardation. This effect of quercetin on the development of preimplantation embryos may be related to the antioxidant actions of quercetin in reducing ROS levels in zygotes.

It is well known in biological systems that oxidative damage may result from a high concentration of ROS, which consequently induces apoptotic cell death in various cell types [37]. Previous research showed that supplementation of quercetin in culture media effectively prevented H_2_O_2_-induced ROS accumulation and inhibited apoptotic signals in P19 neuron cells [38]. Moreover, quercetin also markedly suppressed apoptosis in H9c2 cardiomyoblast cells through the reduction of intracellular ROS [39]. Consistently, our data also showed that quercetin exposure not only prevented the increase in intracellular ROS induced by H_2_O_2_, but also significantly decreased the number of apoptotic cells and increased the total cell numbers in blastocysts. This effect of quercetin may be attributed to its polyphenolic structure to balance intracellular ROS [20].

Aside from quenching ROS directly, maintaining stable mitochondrial function also seems to contribute to the powerful antioxidant properties of quercetin. During early embryo development, Mitochondrial dysfunction may contribute to both cell cycle arrest and apoptosis [11]. As an important parameter of mitochondrial function, MMP has been used as an indicator of cell apoptosis [40]. In current research, we found that administration of H_2_O_2_ led to a dissipation of MMP and this effect could blocked by pre-incubation with quercetin, which is consistent with the result as previous study [41].

In embryogenesis, cytosolic GPx, an enzymatic antioxidant, is highly expressed in most cells and tissues and protects the embryos from oxidative stress [42]. In addition to GPx, CAT is another enzymatic antioxidant expressed in embryos and a vital role for CAT as an antioxidant has been demonstrated through knock-out and transgenic approaches in mice [43]. Our study found that quercetin not only strongly rescued the T-AOC and activities of GPx to similar levels of the control under oxidative stress, but also partially recovered CAT activity, which suggests that quercetin might protect mouse embryos against ROS by sparing antioxidative enzymes.

In addition to enzymatic defenses, glutathione, a non-enzymatic antioxidative defense and detoxifies hydrogen peroxide via action of GPx, plays a prominent role in maintaining the redox status in embryos [5]. Although previous studies have reported that quercetin has the capability to keep intracellular GSH at high levels [44], [45], we did not find any significant difference in GSH levels between the groups in the presence or absence of quercetin.

Intriguingly, depending on the dose and experimental situation, quercetin has conflicting roles – antioxidant or prooxidant. In Jurkat T-lymphocytes, low concentrations of quercetin protected cellular DNA from oxidant injury, while high concentrations of quercetin had the opposite effect [46]. Consistently, the current study found that 50 uM quercetin significantly decreased early embryo developmental potential, acting as a prooxidant. As well, 10 μM quercetin treatment showed a slight decrease in hatched rate of implantation embryos as compared with the control, but when the concentration decreased to 5 μM, a protective effect of quercetin on embryos development was observed. These data suggested that the antioxidative function of quercetin is dose-sensitive.

As a vehicle for the flavonoids, it has been reported that 0.1% DMSO, had significant protection on the mouse embryos owning somites from the oxidative stress [47]. Our data showed that 0.01% DMSO, the concentration used for dissolving quercetin, had no protective effect on early embryo development. First, quercetin alone treatment (dissolved in DMSO), at 1 μM, 5 μM, 10 μM, showed no effect on embryo development potential. Second, 0.01% DMSO also had no protection on early embryo development under oxidative stress. In contrast to the report from Perez-Pasten R [47], 0.1% DMSO treatment enhanced the oxidative damage on the early embryo development, which might due to the distinct developmental stage.

According to the species and developmental stage, embryos may have different sensitivities to oxidative stress. For example, Morales *et al.* have shown that the development potential of bovine zygotes and blastocysts exposed to H_2_O_2_ were compromised in a dose-dependent manner, whereas the 9- to 16-cell embryos were unaffected by this treatment [16]. Our research also showed that exposure of zygotes to 100 μM H_2_O_2_ dramatically decreased the hatched rate to 0% (data not shown). Additionally, the zygote stage is also responsive to the antioxidative effect of quercetin. Moreover, *in vitro* culture systems, zygotes develop in an environment lacking confounding signaling from neighboring cells [11]. Therefore, the antioxidant function of quercetin might be exerted autonomously through the zygote's own defense system rather than cumulus cells or tube fluids.

In summary, our results showed that under oxidative stress, mouse zygotes exposed to quercetin maintained stable mitochondrial function, increased T-AOC, GPx and CAT activities and decreased ROS levels, thereby improving embryo quality and the rate of development *in vitro*. These results introduce a potential improvement in the method to prevent oxidative damage to preimplantation embryos.

## Supporting Information

Table S1 **Development of preimplantation mouse embryos after treatment with H_2_O_2_.**
(DOC)

Table S2 **Development of preimplantation mouse embryos in the prescence of quercetin.**
(DOC)

Table S3 **Effect of DMSO on the development of preimplantation mouse embryos under H_2_O_2_-induced** **stress.**
(DOC)
