# Supplementary material for: Protective Effect of Quercetin on the Development of Preimplantation Mouse Embryos against Hydrogen Peroxide-Induced Oxidative Injury
Source: PLoS One. 2014 Feb 21;9(2):e89520. doi: 10.1371/journal.pone.0089520 (PMC3931787; doi:10.1371/journal.pone.0089520)
Supplement: Table S1 — Development of preimplantation mouse embryos after treatment with H2O2. (DOC) [file pone.0089520.s001.doc]

**Table S1. Development of preimplantation mouse embryos after treatment with H_2_O_2_**.

|  |  |  | **Zygotes developing to: n (% of A)** | | | |
| --- | --- | --- | --- | --- | --- | --- |
| **Groups** | **Conc. of H_2_O_2_ (μM)** | **No. of zygotes (A)** | **Two-cell stage** | **Morula stage** | **Blastocyst stage** | **Hatched stage** |
| Control | 0 | 109 | 108(99.083) | 102(93.578) | 89(81.651) | 62(56.881) |
| Treatment | 20 | 85 | 85(100) | 81(95.294) | 69(81.176) | 43(50.588) |
|  | 35 | 87 | 87(100) | 70(80.46) | 52(59.77)^**^ | 32(36.872)^**^ |
|  | 50 | 59 | 45(76.271)^***^ | 34(57.627)^***^ | 27(45.763)^***^ | 23(39.983)^***^ |
|  | 75 | 42 | 19(45.238)^***^ | 18(42.857)^***^ | 14(33.333)^***^ | 9(21.429)^***^ |

Differences between the groups were calculated using the *x*^2^-test.

^**^ *P*<0.01, ^***^ *P*<0.001 vs. the control group.
