# Supplementary material for: Protective Effect of Quercetin on the Development of Preimplantation Mouse Embryos against Hydrogen Peroxide-Induced Oxidative Injury
Source: PLoS One. 2014 Feb 21;9(2):e89520. doi: 10.1371/journal.pone.0089520 (PMC3931787; doi:10.1371/journal.pone.0089520)
Supplement: Table S2 — Development of preimplantation mouse embryos in the prescence of quercetin. (DOC) [file pone.0089520.s002.doc]

**Table S2. Development of preimplantation mouse embryos in the prescence of quercetin.**

|  |  |  | **Zygotes developing to: n (% of *A*)** | | | |
| --- | --- | --- | --- | --- | --- | --- |
| **Groups** | **Conc. of Que (μM)** | **No. of zygotes (*A*)** | **Two–cell stage** | **Morula stage** | **Blastocyst stage** | **Hatched stage** |
| Control | 0 | 83 | 83(100) | 73(87.952) | 67 (80.723) | 36(43.373) |
| Treatment | 1 | 82 | 82(100) | 73(89.024) | 65(79.268) | 36(43.902) |
|  | 5 | 83 | 83(100) | 71(85.542) | 67(80.723) | 37(44.578) |
|  | 10 | 83 | 83(100) | 70(84.337) | 60(72.289) | 25(30.120) |
|  | 50 | 69 | 68(98.551) | 28(40.580)^***^ | 10(14.493)^***^ | 2(2.899)^***^ |

Differences between the groups were calculated using the *x*^2^-test. Que: quercetin.

^***^ *P*<0.001 vs. the control group;
