# Supplementary material for: Protective Effect of Quercetin on the Development of Preimplantation Mouse Embryos against Hydrogen Peroxide-Induced Oxidative Injury
Source: PLoS One. 2014 Feb 21;9(2):e89520. doi: 10.1371/journal.pone.0089520 (PMC3931787; doi:10.1371/journal.pone.0089520)
Supplement: Table S3 — Effect of DMSO on the development of preimplantation mouse embryos under H2O2-induced stress. (DOC) [file pone.0089520.s003.doc]

**Table S3. Effect of DMSO on the development of preimplantation mouse embryos under**

**H_2_O_2_-induced stress**

|  |  |  |  | **Zygotes developing to: n (% of A)** | | | |
| --- | --- | --- | --- | --- | --- | --- | --- |
| **Groups** | **Conc. of H_2_O_2_ (μM)** | **Conc. of DMSO (%)** | **No. of zygotes (A)** | **Two–cell stage** | **Morula stage** | **Blastocyst stage** | **Hatched stage** |
| Control | 0 | 0 | 123 | 118(95.935) | 110 (89.431) | 99 (80.488) | 61(49.593) |
| H_2_O_2_ | 35 | 0 | 128 | 122(95.313) | 89(69.531)^***^ | 70(54.688)^***^ | 34(26.563)^***^ |
| H_2_O_2_+DMSO | 35 | 0.1 | 123 | 118(95.935) | 52 (42.276)^***，###^ | 51(41.463)^***，#^ | 28(22.764)^***^ |
|  | 35 | 0.01 | 124 | 121(97.581) | 78(62.903)^***^ | 78 (62.903)^**^ | 40(32.258)^**^ |

Differences between the groups were calculated using the *x*^2^-test.

^**^*P*<0.01, ^***^ *P*<0.001 vs. the control group;

^#^ *P*<0.05, ^###^ *P*<0.001 vs. the H_2_O_2_-treated group.
